# Attenuating the emergence of anti-fungal drug resistance by harnessing synthetic lethal interactions in a model organism

**DOI:** 10.1371/journal.pgen.1008259

**Published:** 2019-08-19

**Authors:** Jane Usher, Ken Haynes

**Affiliations:** Biosciences, University of Exeter, Exeter, United Kingdom; University of Georgia, UNITED STATES

## Abstract

Drug resistance is a rapidly emerging concern, thus prompting the development of novel therapeutics or combinatorial therapy. Currently, combinatorial therapy targets are based on knowledge of drug mode of action and/or resistance mechanisms, constraining the number of target proteins. Unbiased genome-wide screens could reveal novel genetic components within interaction networks as potential targets in combination therapies. Testing this, in the context of antimicrobial resistance, we implemented an unbiased genome-wide screen, performed in *Saccharomyces cerevisiae* expressing a *Candida glabrata PDR1+* gain-of-function allele. Gain-of-function mutations in this gene are the principal mediators of fluconazole resistance in this human fungal pathogen. Eighteen synthetically lethal *S*. *cerevisiae* genetic mutants were identified in cells expressing *C*. *glabrata PDR1+*. One mutant, lacking the histone acetyltransferase Gcn5, was investigated further. Deletion or drug-mediated inhibition of Gcn5 caused a lethal phenotype in *C*. *glabrata* cells expressing *PDR1^+^* alleles. Moreover, deletion or drug-mediated inactivation of Gcn5, inhibited the emergence of fluconazole-resistant *C*. *glabrata* isolates in evolution experiments. Thus, taken together, the data generated in this study provides proof of concept that synthetically lethal genetic screens can identify novel candidate proteins that when therapeutically targeted could allow effective treatment of drug-resistant infections.

## Author summary

Life threatening infections are an increasing reality. Multi-drug resistant bacteria *e*.*g*. MRSA are present in nearly all hospitals, and community acquired TB is often recalcitrant to treatment. Less well known, but causing four to five times more deaths in the UK than MRSA, are fungi in the genus *Candida*. These are commonly associated with mucosal infections such as “thrush”, but are responsible for > 400,000 life-threatening infections worldwide every year, especially in the immunosuppressed patient population. One of the principle pathogens is *Candida glabrata*. This fungus grows as a single celled yeast, and alarmingly is highly resistant to both host innate defences and many clinically used antifungal drugs. Even with the best possible medical care, infections with this yeast cause ~50% mortality and alarmingly the incidence of *C*. *glabrata* infections is steadily climbing. This demands that novel antifungal therapies are developed that can target drug resistant fungal infections such as *C*. *glabrata*. We have identified genes that prevent the growth of fungal cells that express gain-of-function mutations in Pdr1, a key mediator of antifungal resistance in *C*. *glabrata*. We also provide proof of concept that targeting a protein essential for Pdr1 function significantly inhibits the emergence of antifungal drug resistance. Thus such an approach could provide a powerful tool in developing treatments for drug resistant infections.

## Introduction

Drug resistance has emerged as a huge problem in many areas of medicine from cancer to infectious diseases [1, 2, 3, 4]. This is leading to the development of novel therapeutic strategies. Multi-target therapies are gaining ground, where combinations of drugs targeting different components of disease networks are deployed with the expectation of reduced toxicity, emergence of resistance, and off-target effects [5, 6, 7]. Combinatorial therapies involving an antibiotic and a second drug either targeting the same pathway, another cellular function, and/or specific mechanisms of antimicrobial drug resistance have shown promise as therapeutic regimens to treat antimicrobial drug resistant infections [8]. A major impediment to this approach is the characterization of the adjunctive targets. To date most adjunctive therapy targets have been selected based on previous biological knowledge of drug mode of action and/or mechanisms of resistance. This severely constrains the number of proteins that can be targeted for adjunctive therapy. In this study, we hypothesized that unbiased genome-wide screens can reveal previously unknown proteins that could be targeted for adjunctive therapy. This has recently been demonstrated in the context of cancer, where Cas9 mediated genome editing was used to target chromatin regulatory domains in a murine acute myeloma cell line, identifying six known drug targets, and a further 19 genes that are essential in this cancer cell line [9]. In relation to antimicrobial resistant infection, we rationalised that the characterization of mutations that genetically interact with alleles conferring drug resistance could reveal novel proteins that could be therapeutically targeted to allow effective treatment of antimicrobial drug resistant infections.

Fungi are important agents of infectious disease, causing more deaths annually than either malaria or TB [10]. In this context, *Candida* species are the fourth most commonly isolated species from nosocomial blood stream infections, causing life threatening disease in individuals with AIDs, patients recovering from surgical procedures or major burns, and those undergoing chemotherapy and organ transplant. Systemic fungal infections are currently very difficult to diagnose, and even with best practice management mortality rates are generally higher than for bacterial disease [11]. Furthermore, the effectiveness of the drugs used to treat fungal infections is decreasing, as antifungal drug resistance is rapidly emerging. Antifungal resistance has been reported in environmental fungal isolates suggesting a reservoir of resistant strains [12,13]. There is an urgent clinical and economic need for new cost effective treatment options, including novel therapeutics.

*Candida glabrata* ranks second after *Candida albicans* as the most common yeast pathogen of humans. It is responsible for many opportunistic infections in immunocompromised individuals, which are associated with a high mortality rate. The incidence of *C*. *glabrata* infections has grown rapidly over the last 20 years, and is responsible for ~25% of systemic candidiasis cases [14,15]. The reason for this increasing incidence of *C*. *glabrata* infection is not fully understood, but it is well established that this species has a higher innate tolerance to commonly administered azole antifungals, in particular fluconazole (FLZ), the principle therapeutic option for *Candida* infections. For instance, *C*. *glabrata* populations have an average MIC to fluconazole (FLZ) of 4 μg/ml compared to 0.125 μg/ml for *C*. *albicans* populations [16–18]. Alarmingly, *C*. *glabrata* is also adept at rapidly acquiring drug resistance [19]. MIC values of 64 μg/ml are found in up to 30% of *C*. *glabrata* isolates, and thus are often resistant to FLZ therapy [20,21].

**Fig 1:**
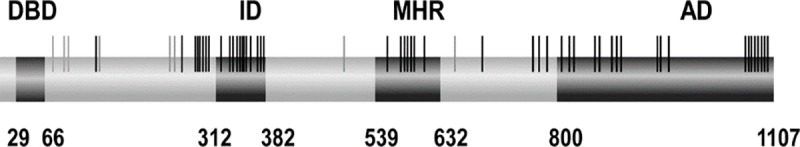
Schematic of PDR1 (CAGL0A00451g). The 4 different domains are indicated by the dark grey boxes – DBD – DNA binding domain; ID – inhibitory domain; MHR- middle homology region and AD – activation domain. Each of the individual light grey and black vertical lines represents a *PDR1* gain of function mutation that has been identified in a clinical isolates resulting in resistance and/or tolerance to azoles.

One of the principle mediators of FLZ resistance and acquired resistance are gain of function mutations in the *PDR1* (CAGL0A00451g) gene (*PDR1^+^*), which encodes a transcriptional activator of genes encoding drug efflux pumps [22]. To date, many *PDR1^+^* mutations have been described that mediate azole resistance (Table 1). These *PDR1^+^* mutations cause amino acid changes across all four functional domains of the transcription factor: the transcriptional activation domain, the regulatory domain, the middle homology region, and the activation domain (Fig 1). *PDR1* is up-regulated during systemic infections [23,24], and is induced in response to combinatorial stresses encountered *in vivo*. *C*. *glabrata* strains harbouring *PDR1^+^* mutations exhibit increased virulence [18] implying adaptation within the host to antifungals may itself enhance the ability of *C*. *glabrata* to cause disease. In this study, we have performed an unbiased genetic screen to identify mutants that are synthetically lethal with *PDR1^+^* fluconazole resistant *C*. *glabrata* cells, having adopted the approach of a combination of genome-wide screens [25] and mutant construction to identify *C*. *glabrata* loss of function mutations that interact to impact negatively on the growth in combination of with specific FLZ resistant alleles. We then used prior knowledge to identify which of the proteins encoded by these genetic interactors can be targeted therapeutically, either using known or newly discovered small molecule inhibitors, to treat FLZ resistant *C*. *glabrata*.

**Table 1:** PDR1 clinical isolates and location of gain-of-function mutation used in this study.

Clinical Isolate name	PDR1 allele
DSY486	WT*
DSY489	L328F
**DSY562**	WT*
**DSY565**	**L280F**
DSY2253	WT*
DSY2254	D1082G
DSY529	WT*
DSY530	E1083Q
DSY738	WT*
DSY739	R376W
DSY726	WT*
DSY727	D876Y
DSY753	WT*
DSY754	Y584C
DSY2234	WT*
DSY2235	T607S
DSY3629	P822L
DSY2726	F853Q
DSY2746	I373V
DSY2725	Y285C
DSY1169	V785D
DSY1180	D1089Y
DSY2257	N691D
DSY2268	S316I
DSY1185	R592G
DSY2279	G583S
DSY2271	D261G
DSY2273	R293I
DSY756	S343F
DSY2315	R376G
DSY2277	R592S

* refers to the parental strain of gain-of-function mutations that arose after FLZ exposure.

Mutations are termed ‘synthetically lethal’ if either mutation alone has no impact on cellular viability, but in combination result in cellular death. Our hypothesis, was that by inhibiting the product of a gene whose deletion is synthetically lethal with *PDR1^+^* alleles will allow targeting of FLZ resistant *C*. *glabrata*. To test this prediction we set to address two questions; what are the synthetic lethal/synthetic sick interaction partners of *PDR1^+^* alleles, and, as proof of concept, can any of these be targeted to prevent the emergence of azole resistant *C*. *glabrata*? Therefore the primary aim of this work is to identify pathways that could be targeted to prevent the emergence of antifungal drug resistance. In this study we have taken this methodology to identify conserved synthetic genetics interactions across a *PDR1^+^* allele, then used this data to identify small molecule inhibitors of these synthetic interactors (namely GCN5 inhibitor ɣ-butyrolactone) and determined if they can be used to treat FLZ resistant *C*. *glabrata*.

## Results & discussion

### SDL-SGA screen of *C. glabrata PDR1^+^*

**Fig 2:**
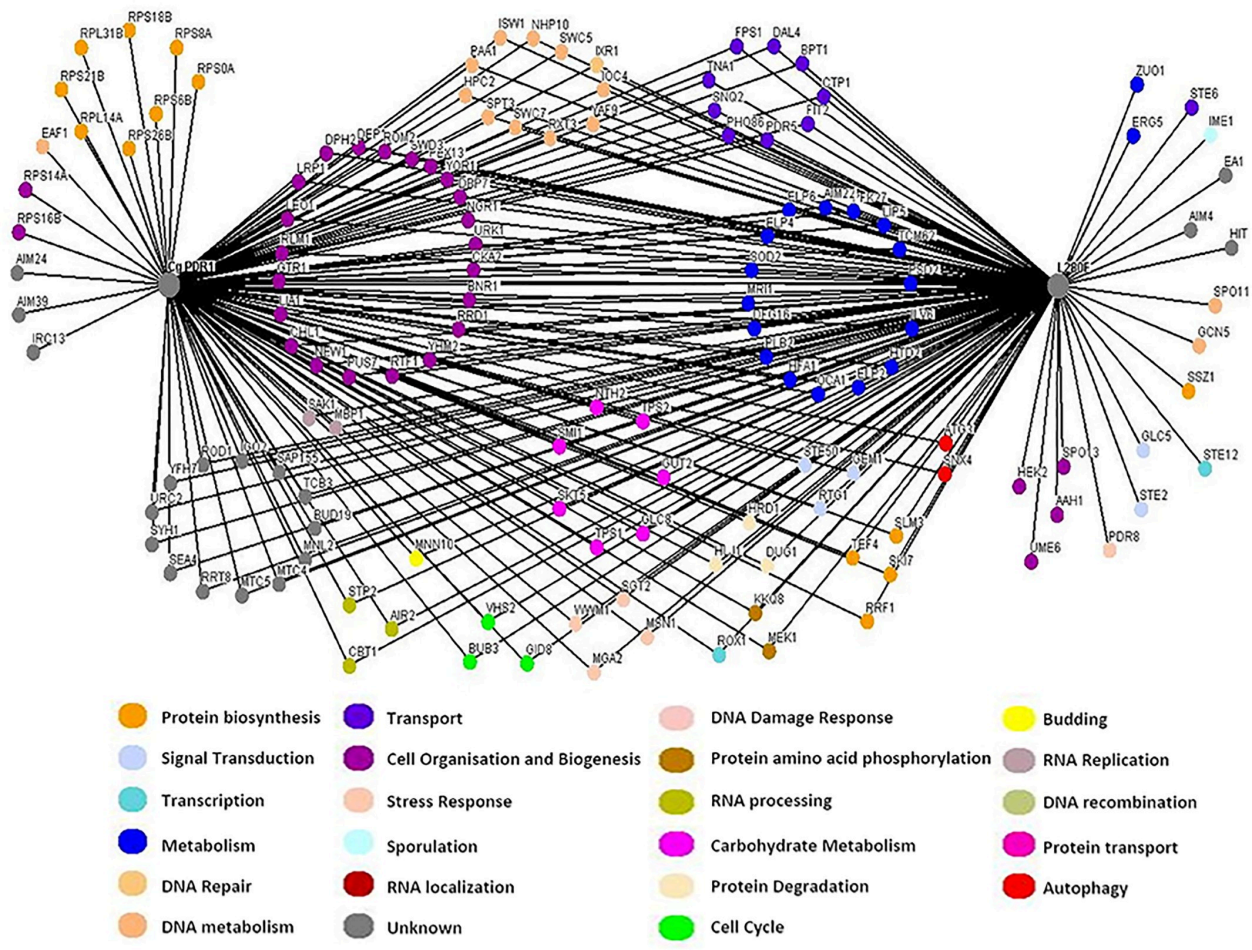
Genetic interaction network map of C. glabrata PDR1^WT^ and PDR1^L280F^. Genome-wide synthetic interaction SGA screens were performed using query strains expressing either the wild type or *PDR1*^*+L280F*^ *C*. *glarbata* ORF. Genes are represented by nodes that are colour coded corresponding to their cellular roles (www.yeastgenome.org and www.candidagenome.org) and/or assigned through review of the literature. Interactions are represented by edges. A comprehensive list of all interactions can be found in the supplementary information.

Synthetic Genetic Array (SGA) screening is not currently possible in *C*. *glabrata*, as the technique relies on high throughput mating. Hence to identify *PDR1^+^* synthetic genetic interactions, we used the model yeast *S*. *cerevisiae* as a surrogate with a view that key synthetic lethal interactions would subsequently be confirmed in *C*.*glabrata*. To initiate the characterization of *C. glabrata PDR1^+^* synthetic genetic interaction network, we performed a synthetic dosage lethal (SDL)-SGA experiment [26], to identify synthetic interactions with a *PDR1^+L280F^* allele described in a clinical *C*. *glabrata* isolate DYS565 (Fig 2) [19,21]. This particular allele was chosen due to its poor clinical outcomes and high FLZ MIC [27]. The DYS565 strain has a FLZ MIC of 128 μg/ml and a G840C (L280F) mutation (*PDR1^+L280F^*), whereas the parental strain, DYS562, obtained from the same patient is relatively FLZ sensitive (MIC 8 μg/ml) and contains a wild-type *PDR1* allele. The *PDR1^+L280F^* was amplified by PCR, sequence verified, cloned and transformed into an *S*. *cerevisiae MAT***a** SGA starter strain, with the endogenous copy of *PDR1* deleted and then mated to the entire *MAT*α knock-out collection. This genome-wide SGA screen was performed in triplicate and all double mutants were visually scored for growth. A total of 144 negative genetic interactions were identified with the *C. glabrata PDR1^+L280F^* allele (Fig 2, S1 Table), of which 22 were synthetically lethal (S2 Table) and the remainder caused significant reductions in growth. Of the 22 synthetic lethal interactions four were also lethal with wild-type *PDR1* i.e. *elp2*, *elp4*, *elp6* and *pdr5*. Elp2, Elp4 and Elp6 are all components of the elongator complex, while Pdr5 is a multidrug transporter involved in pleiotropic drug responses. Thus 18 strains had specific lethal interactions with *C. glabrata PDR1^+L280F^*and included genes with functions related to drug transport (*ERG5*, *EAF1*), and others transcription factors (e.g. *PDR3*, *PDR8*, *STE12* and *UME6*). In the case of the synthetic sick interactions identified in both screens, CgPDR1 (104 interactions) and *PDR1^+L280F^* (105 interactions), 90 were common to both screens with 14 unique to CgPDR1 and 15 unique to *PDR1^+L280F^* (S1 Table and Fig 2).

**Fig 3:**
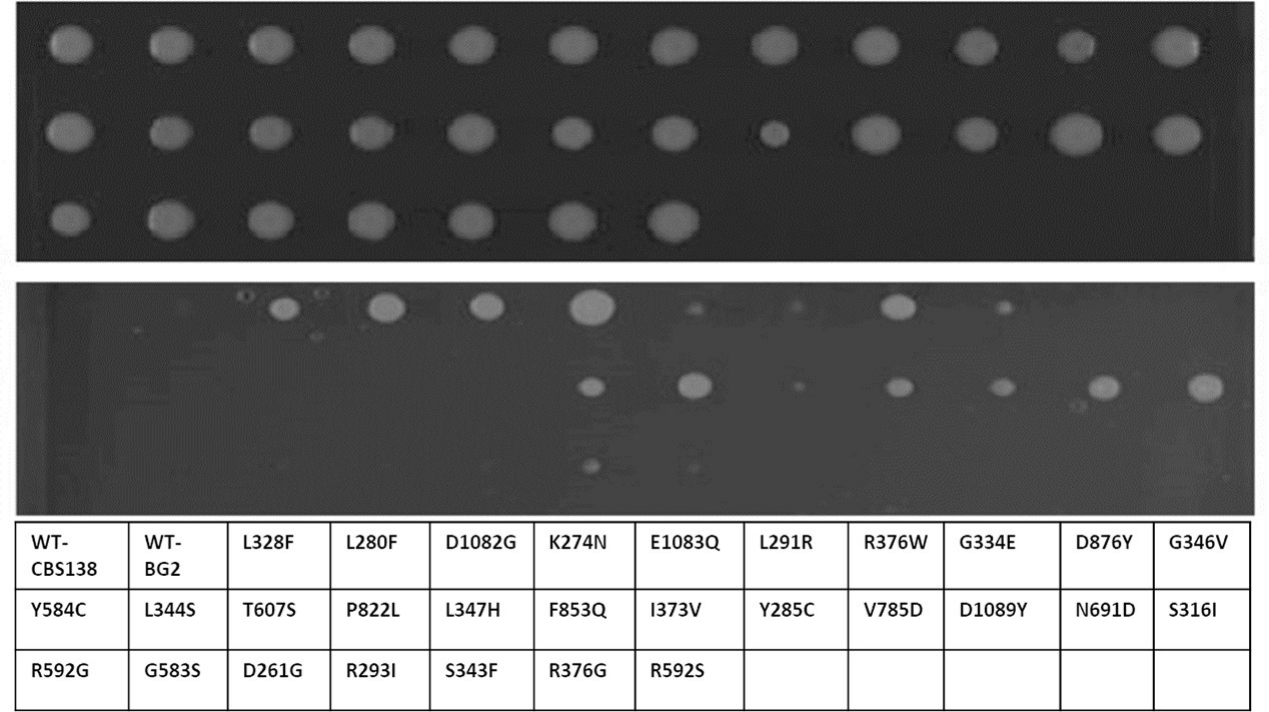
FLZ resistant clinical isolates of *C*. *glabrata* containing *PDR1*^*+*^ alleles are sensitive to ɣ-butyrolacetone. *C*. *glabrata* FLZ resistant clinical isolates containing *PDR1*^*+*^ mutations were grown on synthetic complete (top panel) or synthetic complete +2mM ɣ-butylroacetone (bottom panel). The ɣ-butylroacetone was synthetic lethal to 20/31 FLZ resistant *C*. *glabrata* clinical isolates containing *PDR1*^*+*^ alleles. Plate layout showing which gain-of-function mutants screened shown below the gel images.

To determine if these genetic interactions were maintained with different *PDR1*^+^ gain of function alleles, we performed tailored SGA screens. Specifically, the previous interactions identified from the *PDR1^+L280F^* screen were sub-arrayed to determine if the synthetic lethal interactions were common to other gain of function alleles. Four other gain-of-function alleles were selected; S316I, L1391I, E555K and F817S, covering the four main functional domains of Pdr1 (S3 Table, S1 Fig and Fig 1). From this refined screen, we were confident in following GCN5 as our proof of principle gene of interest for chemical inhibition, due to its synthetically lethal interaction with the additional gain of function mutants screens and when chemically inhibited in a series of gain of function mutants we were able to induce lethality in the strains (Fig 3).From these additional screens, we identified 9 SL interactions that were common to all gain of function genes tested; *DUG1*, *EA1*, *ELP4*, *GLC5*, *HEK2*, *PDR5*, *PDR8*, *STE12 and STE2*. These genes offer potential further targets for chemical inhibition in future studies, across a variety of molecular functions. In *S*. *cerevisiae GCN5* encodes a component of the ADA and SAGA histone acetyltransferase complexes.

### Deletion of *GCN5* is synthetically lethal in *C. glabrata PDR1^+^* cells

To test our hypothesis that drug targeting of lethal interactors, identified above, would abolish survival of drug resistant *PDR1^+^* cells, we focussed on *GCN5* for two reasons. Firstly, deletion of *GCN5* was synthetically lethal with the five gain-of-function *PDR1^+^* alleles tested and, secondly, there is a well-characterized specific inhibitor of the Gcn5 HAT, γ-butyrolactone. Thus, if our hypothesis is correct, prevention of Gcn5 function through γ-butyrolactone treatment, should kill *C*. *glabrata* cells harbouring the drug resistant *PDR1^+L280F^*allele.

**Fig 4:**
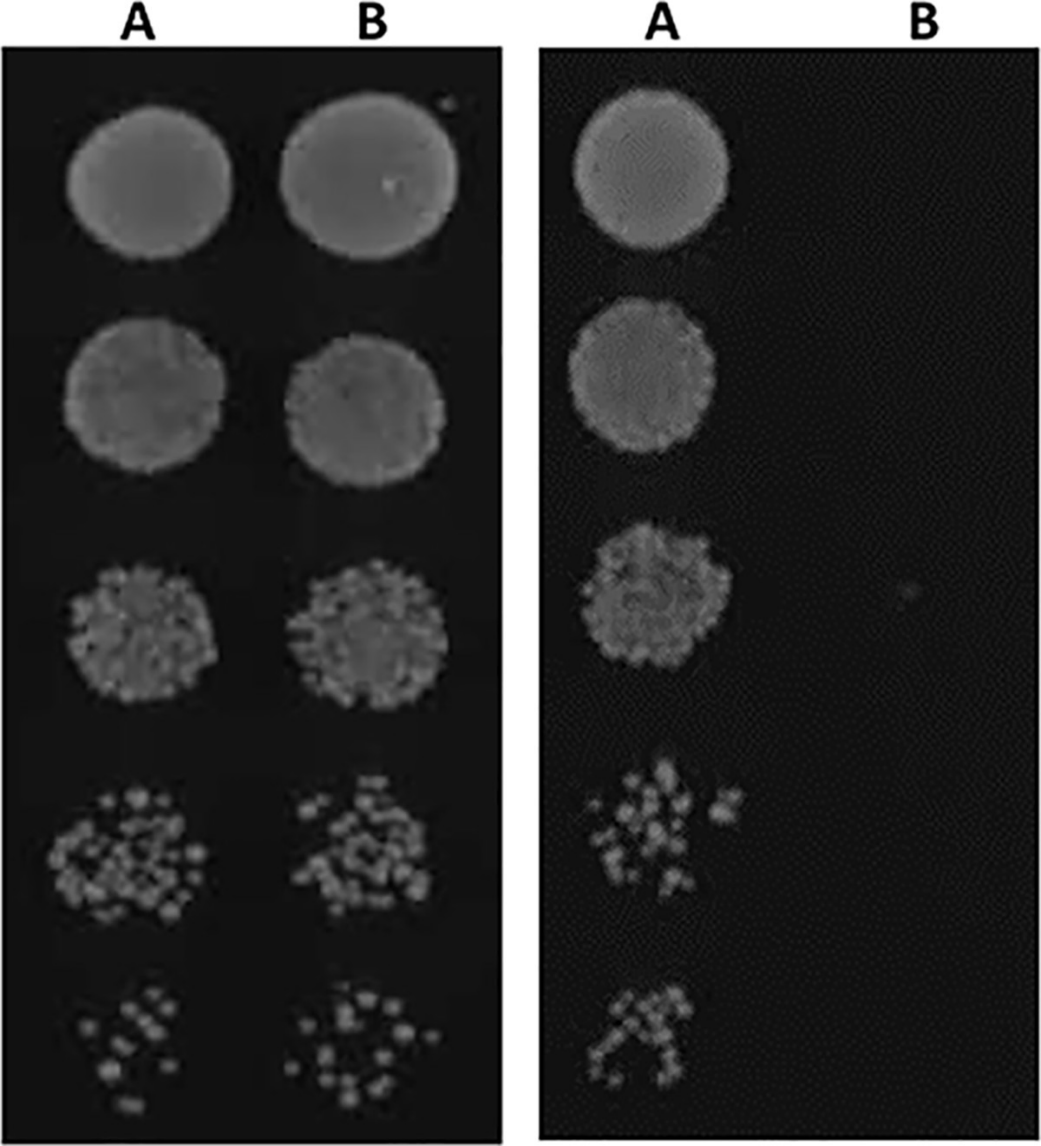
Confirmation that expression of *PDR1*^*+L280F*^ in *C*. *glabrata gcn5Δ cells* is synthetically lethal. A *C*. *glabrata gcn5 pdr1* strain was transformed with pCU-MET3 (A) or pCU-MET3 containing PDR1^L280F^ (B) and cultured on synthetic complete media (left panel) or synthetic complete media lacking methionine (right panel). Induction of *PDR1*^*L280F*^ is lethal in *C*. *glabrata gcn5* cells thus confirming the synthetic lethal interaction identified in *S*. *cerevisiae*.

As a first step, we confirmed that expression of *PDR1^+L280F^* in a *C*. *glabrata gcn5* null mutant background was lethal (Fig 4). To achieve this *PDR1^+L280F^* was placed under the control of the methionine repressible promoter in pCU-MET3 [28] and transformed into a *C*. *glabrata gcn5 pdr1* double null mutant. The induction of *PDR1^+L280F^* in this strain resulted in a loss of viability, thus confirming that the synthetic lethal interaction identified in *S*. *cerevisiae* is conserved in *C*. *glabrata*.

Once we had confirmed that loss of Gcn5 function is lethal in *C*. *glabrata* cells expressing the *PDR1^+L280F^* fluconazole resistant allele, we then determined the impact of chemically inhibiting Gcn5. Notably, the addition of 2mM γ-butyrolactone, the chemical inhibitor of Gcn5, prevented the growth of *S*. *cerevisiae pdr1Δ* strains expressing *C. glabrata PDR1^L280F^*, the clinical FLZ resistant *C*. *glabrata* strain DYS565 expressing *PDR1^L280F^*, and an engineered *C*. *glabrata* lab strain (BG2 derivative) in which the wild-type *PDR1* allele was replaced with *PDR1^L280F^* (Fig 4). Collectively, these data demonstrate that targeting Gcn5, a synthetically lethal interacting partner of *PDR1^L280F^* identified in *S*. *cerevisiae*, renders both this species and the orthologous *C*. *glabrata* mutant inviable, strongly supporting the proposition that targeting synthetic lethal interactions offers a new paradigm for the treatment of drug resistant infection.

To further explore this concept, we investigated whether the addition of ɣ-butyrolactone would prevent and/or inhibit the growth of addition FLZ-resistant clinical isolates with different gain of function mutations in *PDR1* (Table 1). Notably, ɣ-butyrolactone prevented the growth of 20/31 clinical isolates screened (Fig 4). This demonstrates that the chemical inactivation of the Gcn5 protein is synthetically lethal in approximately 65% of the *PDR1^+^* FLZ resistant alleles tested.

### Does the deletion of synthetic lethal genes, or chemical inactivation of their encoded proteins reduce the emergence of FLZ resistance in *C*. *glabrata*?

Finally, we examined whether targeting synthetic lethal interactions could minimise the emergence of FLZ resistance utilising an experimental evolution approach [29]. Using such an approach allowed for the observation of the impact of their deletion on the emergence of FLZ resistance. *C*. *glabrata* wild-type and *gcn5* null strains, together with wild-type cells in which the function of Gcn5 was chemically inhibited, were exposed to doubling dilutions of FLZ and the emergence of resistance monitored. Our working hypothesis was that FLZ resistance would emerge at a much-reduced rate and to a lower level in strains that had synthetic lethal genes deleted or chemically inactivated (in this case *GCN5*), compared to wild-type *C*. *glabrata*.

**Fig 5:**
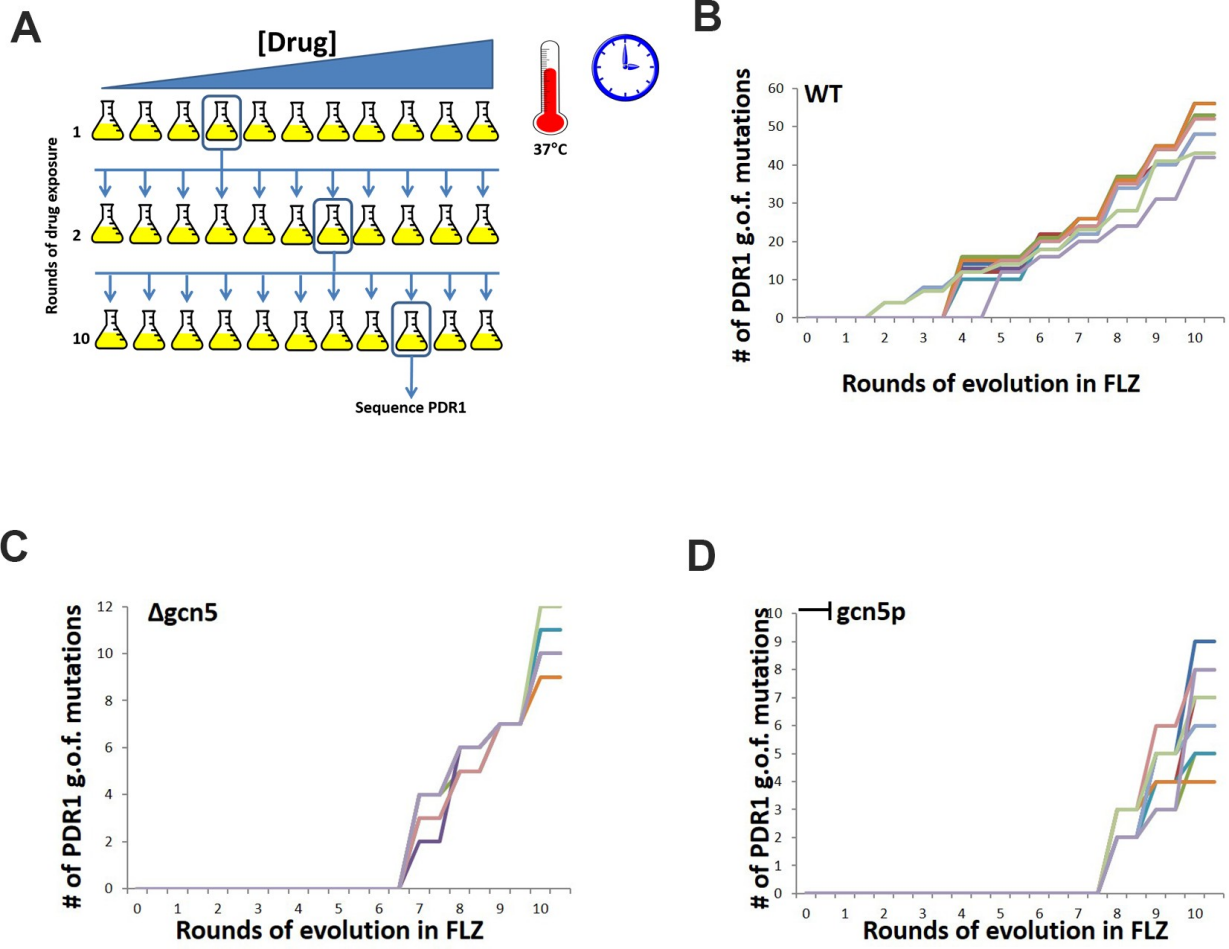
Evolution of wildtype, gcn5 null and gcn5 chemically inhibited *C*. *glabrata* cells in the presence of fluconazole. *C*. *glabrata* cells grown in increasing concentrations of FLZ, with the PDR1 gene sequenced after each round to determine when and in which domain gain of function mutations are identified in. (**A) Schematic of experiment.** 10 individual flasks of *C*. *glabrata* cells – wildtype, Δgcn5 and chemically inhibited Gcn5 were exposed to increased concentrations of FLZ. The flask where inhibition of growth was first observed was used as the started culture for the subsequence round of drug exposure until 10 rounds of drug exposure was completed. At each pitching of cells, PDR1 was sequenced to identified when a gain of function mutation first emerged. (**B)** In wildtype *C*. *glarbata* cells, after 3 rounds of exposure to increasing FLZ concentrations, PDR1 mutations were identified and mapped to the activation domain. (**C)** *C*. *glabrata* Δgcn5 cells, after 6 rounds of exposure to increasing FLZ concentrations, PDR1 gain of function mutations were isolated and mapped to the activation domain and the middle homology domain. (**D)** *C*. *glabrata* cells that had Gcn5p chemically inhibited through with the addition of ɣ-, after 7 rounds of exposure to increasing FLZ concentrations, the emergence of PDR1 gain of function mutations was observed in the activation domain and DNA binding domain. The number of gain of function mutations observed is dramatically reduced in both the Δgcn5 and chemically inhibited Gcn5 FLZ exposures.

As each propagation was made to the next round of selection, the *PDR1* gene was sequenced to determine in each condition, and at which cycle, gain of function mutations started to arise in the populations and to what region of the gene they mapped to. For the wild type strain BG2, after going through three rounds of exposure to FLZ and up to a concentration of 8μg/ml, we identified the appearance of the first gain of function mutation in the *PDR1* allele (Fig 5A). This mutation was located in the activation domain of the gene. As the exposure to FLZ continued for a further 7 cycles, there was a noted increase in the number of gain of function mutations arising in the wild type strain. This was also linked to the increase in FLZ concentration. Following the 10 cycles of propagation in FLZ, we had identified 50 previously described gain of function mutations in *PDR1* (Fig 5B).

In the case of the *gcn5* null strain (Fig 5C), and the chemically inhibited *gcn5* strain (Fig 5D), a gain of function mutation was not observed in *PDR1* until 7 rounds of propagation in FLZ. In the *gcn5* null mutant, the T2450C (F817S) mutation in the activation domain (Table 2) was the first observed mutation, whereas T2575 (F859L) was the first mutation identified in the chemically inhibited strain. From this data, it is possible to determine that the evolution of *C*. *glabrata Δgcn5* mutants in the presence of FLZ inhibits the emergence of gain of function mutations in PDR1.

**Table 2:** Gain-of-function mutations in the activation domain of *CgPDR1* observed during evolution in presence of FLZ.

T2450C (F817S)
C2465T (P822L)
T2558C (F853S)
T2575C (F859L)
G2626T (D876Y)
G2827A (G943S)
T2837C (L946S)
T2842A (F948I)
A3229G (N1077D)
G3235A (G1079R)
G3236T (G1079V)
C3239T (T1080I)
A3245G (D1082G)
G3265T (D1089Y)
T3278C (L1093P)
G3296C (G1099A)

### Concluding remarks

In this proof of concept study we have demonstrated that the identification of synthetic lethal genetic interactions with alleles that confer antifungal drug resistance is a valuable approach to identify pathways that could be targeted to prevent the emergence of drug resistance. By employing SGA analysis we identified a number of genetic mutations that were synthetically lethal with *PDR1^+^* gain-of-function alleles. Focussing on one specific mutation, that in the histone acetyltransferase Gcn5, we could show that deletion or chemical inactivation of Gcn5 significantly inhibited the emergence of *PDR1^+^* gain-of-function alleles in evolution experiments. Histone modifications modulate the packing of chromatin, this level of packing is critical for gene transcription, as the cellular machinery must have access to promoters to allow for transcription. As previously stated GCN5 in *S*. *cerevisiae* is known to be a component of the ADA and SGA complexes, therefore we propose that in *C*. *glabrata* clinical isolate with gain of function mutations in PDR1, it is acting as a gene silencer thus resulting in the synthetic lethal phenotype. The combination of the interaction between GCN5 and PDR1^gain of function^ may be resulting in the inhibition of histone acetyltransferases and DNA damage events resulting from drug exposure leading to cell death. This control of chromatin remodelling processes may provide a target for novel drug therapies. Future work employing similar genetic approaches could be powerful in identifying additional targets that could halt the emergence of drug resistant strains.

## Methods

### Strain generation

*PDR1* genes were PCR amplified from their relevant strains, wildtype from BG2 (i.e. no point mutations) and DSY565, the clinical isolate containing the *PDR1^+^* gain of function mutation L280F. PCR products were sequence verified prior to cloning into the Gateway system. Final destination plasmids were transformed into *S*. *cerevisiae* strain Y7092[30], using standard LiAc transformation protocols[31].

### *Candida glabrata* PDR1 synthetic genetic interaction screens

The deletion mutant array was manipulated using a Singer RoTor HAD (Singer Instruments). For the genome wide PDR1 and PDR1^+L280F^ synthetic genetic screens, the MATα query strain Y7092 [30] was transformed with either pDEST426-ccdB-GPD-PDR1 or pDEST426-ccdB-GPD- PDR1^+L280F^. The resulting query strains were mated to the entire MATa deletion mutant array and the SGA methodology was used as previously described to maintain the plasmid [26]. All genome-wide screens were performed in triplicate at 30°C with growth visually scored for lethality (SL), slow growth (synthetic sick SS) or suppression (SUP). Putative genetic interactions were identified in a minimum of two out of three replicates. These putative interactions were then confirmed in *S*. *cerevisiae* and *C*. *glabrata*. (Confirmed genetic interactions are listed in Supporting information, Fig 2).

### Confirmation in *Candida glabrata*

To confirm the SGA screens performed in *S*. *cerevisiae*, we recapitulated the SL phenotypes in *C*. *glabrata*. The *gcn5 pdr1* null mutant was generated using standard deletion protocol [29], followed by transformation of the plasmid containing the PDR1 or PDR1^+L280F^ under the control of the *MET3* promoter [31].

### Dot assays with FLZ and γ-butyrolactone

Dot assays were performed by spotting 5 μl of 10-fold serial dilutions (OD600 = 0.1, 0.01, 0.001, 0.0001) onto specified media, and sealed plates were incubated at 37°C. All dot assay experiments were repeated using three different isolates of each strain. FLZ at concentrations 16–64μg/ml and ɣ-butyrolactone at 2mM were used in screening plates.

### Evolution of *C*. *glabrata* strains in the presence of fluconazole

To examine the effect of FLZ treatment on the genome of *C*. *glabrata* strains, we performed a series of control evolution experiments. We took *C*. *glabrata* strains; BG2, *Δgcn5*, and chemically inhibited BG2, and inoculated into doubling dilution of FLZ from 0-256 μg/ml, in synthetic complete medium at 37°C for 24 hours. The culture at the highest FLZ dose with obvious growth was used to propagate the next FLZ gradient. This was performed for ten cycles with each strain being tested in ten technical replicates. The same workflow was followed for the chemically inhibited gcn5 strain to determine the impact on the emergence of resistance via the addition of γ-butyrolactone to the media. The chemical inhibitor was combined with FLZ. For both experimental regimens the level of FLZ was inferred, for each strain, as the concentration from which propagation was made to the next round of selection. The driving hypothesis for this section of work was that FLZ resistance would emerge at a reduced rate and to a lower level in the strains that have had *gcn5* deleted or chemically inhibited compared to wild-type *C*. *glarbata*.

## Supporting information

S1 Fig Genetic interaction network map of C. glabrata gain-of-function alleles; L139I, E555K, F817S and S316I. Genome-wide synthetic interaction SGA screens were performed using query strains expressing *PDR1*^*+L139I*^, *PDR1*^*+E555K*^, *PDR1*^*+F817S*^ or *PDR1*^*+S316I*^ *C*. *glarbata* ORFs. Genes are represented by nodes that are colour coded corresponding to their cellular roles (www.yeastgenome.org and www.candidagenome.org) and/or assigned through review of the literature. Interactions are represented by edges. A comprehensive list of all interactions can be found in the supplementary S3 Table.
(TIFF)

S1 Table Functional analysis of SGA screens for PDR1 WT and *PDR1*^*+L280F*^. A comprehensive list of interactions from SGA screens for PDR1 wildtype and PDR1 gain-of-function mutation L280F.
(XLSX)

S2 Table SGA analysis for *PDR1*^*+L280F*^. A functional analysis of the genetic interactions for *PDR1*^*+L280F*^. The interactions have been broken down into the separate phenotypic subsets of synthetic lethal and synthetic sick interactions followed by a further breakdown of those unique to the gain-of-function mutation L280F.
(XLSX)

S3 Table Genetic interactions for L139I, E555K, F817S and S316I gain of function mutations in *C*. *glabrata*. Breakdown of the genetic interactins for 4 different gain-of-function mutations in C. glabrata, the interactions are characterised as either SL – synthicaly lethal or SS – synthetically sick phenotype.
(XLSX)
