# Supplementary figures and images for: Attenuating the emergence of anti-fungal drug resistance by harnessing synthetic lethal interactions in a model organism
Source: PLoS Genet. 2019 Aug 19;15(8):e1008259. doi: 10.1371/journal.pgen.1008259 (PMC6715234; doi:10.1371/journal.pgen.1008259)

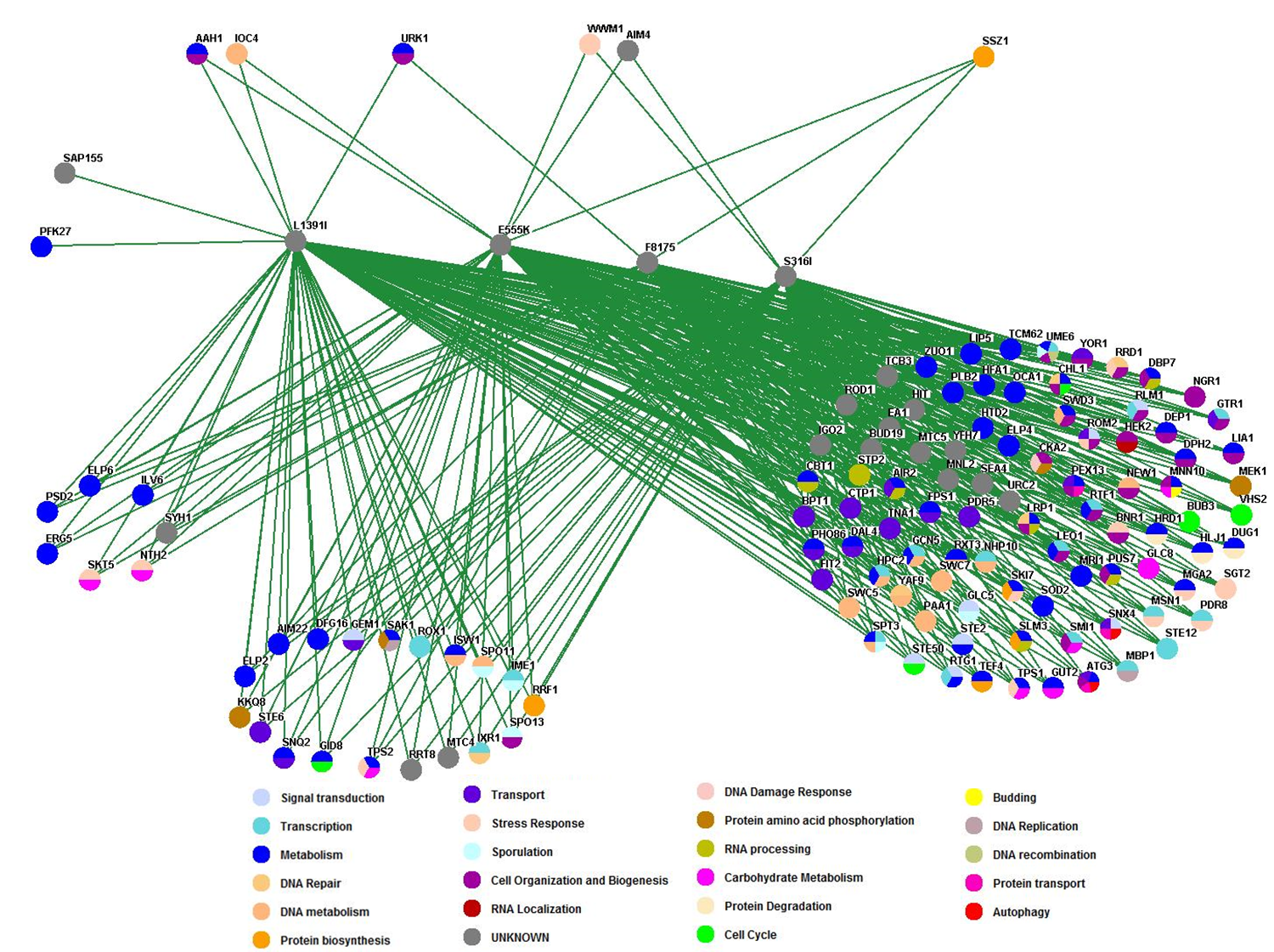

Supplement: S1 Fig — Genome-wide synthetic interaction SGA screens were performed using query strains expressing PDR1+L139I, PDR1+E555K, PDR1+F817S or PDR1+S316I C. glarbata ORFs. Genes are represented by nodes that are colour coded corresponding to their cellular roles (www.yeastgenome.org and www.candidagenome.org) and/or assigned through review of the literature. Interactions are represented by edges. A comprehensive list of all interactions can be found in the supplementary S3 Table. (TIFF) [file pgen.1008259.s001.tiff]
